# How to Limit the Spread of Tuberculosis

**Published:** 1904-11

**Authors:** 


**A Monthly Review of Medicine and Surgery.**

*EDITOR:*

WILLIAM WARREN POTTER, M. D.

All communications, whether of a literary or business nature, books for review and exchanges, should be addressed to the editor: 284 FRANKLIN ST., BUFFALO, N. Y.

VOL. XLIV.—LX. NOVEMBER, 1904. No. 4

### How to Limit the Spread of Tuberculosis.

THE crusade against tuberculosis should be one of the accomplished medical feats of the twentieth century. Our ever-increasing knowledge of its nature—its contagiousness and its virility—give us weapons of great power to wield in the contest of extermination, and if properly and energetically applied, must accomplish the result so eagerly sought. During the past few years the medical profession has become thoroughly organised and massed in the warfare against this dread disease, and now it is time to ask the coöperation and assistance of the laity, and in no way can this be better done than by acquainting the public at large with the knowledge that we have received through years of experience and research.

The one characteristic which above all needs to be emphasised is the contagiousness of the virus, and in no way is the virus disseminated so broadly as in the expectoration of tubercular patients. The sputum of these patients is nothing more than colonies of tubercular bacilli held in solution by the mucus secretions of the upper air chambers. Once dried through exposure to the air the bacilli are freed and their virility being unimpaired they are scattered to the four winds and some assuredly become lodged in the air passages of other individuals where they find a favorable soil for propagation. Thus the army of tuberculotics gains recruits.

Of late there has been an attempt made to cut off this avenue of infection and ordinances have been passed by city authorities, often, however, carelessly enforced, making it a misdemeanor to expectorate in public places. In some cities, Washington and St. Paul notably, expectorating on the sidewalk is punishable by fine or imprisonment, and to the credit of these cities, the ordinances are rigidly enforced.

In Buffalo we have ordinances covering the street cars and public places, but are they ever enforced? Have any fines ever been collected from this source? In many cases the offenders are none other than those paid to enforce the laws.

The habit of expectorating in public is known throughout the world as a habit peculiar to America, and beside being disgusting and filthy is dangerous beyond estimation. Spitting on the sidewalk in front of public places where men and boys are wont to congregate is a public nuisance as objectionable from an ethical and hygienic standpoint as the majority of public nuisances which are punishable and rigidly enforced. Cannot, then, some step be taken to include public spitting with the public nuisances? If so, then cannot the public officials be instructed to carry out the penalties attached to such violations?

It would not be very long if arrests were made and punishment enforced before a very noticeable diminution of this nuisance would be apparent, and one arrest would serve notice to hundreds if not thousands of offenders of the danger they themselves incur by violation of this most wise and humane provision.

